# 

**Published:** 1893-03

**Authors:** 


					﻿Vol XIII.
MARCH, 1893.
NO. 3.
THE
HOMEOPATHIC PHYSICIAN,
A Monthly Journal of Homoeopathic Materia Medica
and Clinical Medicine.
•* He is a freeman whom truth makes free, and all are slaves beside."
"Seek the Truth: come whence it may, cost what it will."
EDITED AND PUBLISHED BY
WALTER M. JAMES, M. D.
PHILADELPHIA :
No. 1125 SPRUCE STJKEHUT.
X» O JST U«O 3V t
ALFRED HEATH & CO., Sole Agents for Great Britain,
114 Ebury Street, Eaton Square.
Yearly Subscription, $2.50. invariably in advance. Single numbers, 25 cts.
Entered at the Philadelphia Poet-Office at Second-Class Mail Matter.
				

